# Seasonal changes in expression of nerve growth factor and its receptors TrkA and p75 in the ovary of wild ground squirrel (*Citellus dauricus* Brandt)

**DOI:** 10.1186/1757-2215-7-3

**Published:** 2014-01-09

**Authors:** Ben Li, Xia Sheng, Lihong Bao, Shiyang Huang, Qinglin Li, Yuning Liu, Yingying Han, Gen Watanabe, Kazuyoshi Taya, Qiang Weng

**Affiliations:** 1 Laboratory of Animal Physiology, College of Biological Science and Technology, Beijing Forestry University, Beijing 100083, China; 2 Institute of Public Health, Inner Mongolia University for Nationalities, Tongliao 028000, China; 3 Department of Veterinary Medicine, Faculty of Agriculture, Laboratory of Veterinary Physiology, Tokyo University of Agriculture and Technology, Tokyo 183-8509, Japan

**Keywords:** Ground squirrel, NGF, Ovary, p75, TrkA

## Abstract

The aim of this study was to investigate the presence of nerve growth factor (NGF) and its receptors tyrosine kinase A (TrkA) and p75 in the ovaries of the wild ground squirrels during the breeding and nonbreeding seasons. In the breeding period, NGF, TrkA and p75 were immunolocalized in granulosa cells, thecal cells, interstitial cells and luteal cells whereas in the nonbreeding period, both of them were detected only in granulosa cells, thecal cells and interstitial cells. Stronger immunostaining of NGF, TrkA and p75 were observed in granulosa cells, thecal cells and interstitial cells in the breeding season compared to the nonbreeding season. Corresponding for the immunohistochemical results, immunoreactivities of NGF and its two receptors were greater in the ovaries of the breeding season then decreased to a relatively low level in the nonbreeding season. The mean mRNA levels of NGF, TrkA and p75 were significantly higher in the breeding season than in the nonbreeding season. In addition, plasma gonadotropins, estradiol-17β and progesterone concentrations were significantly higher in the breeding season than in the nonbreeding season, suggesting that the expression patterns of NGF, and TrkA and p75 were correlated with changes in plasma gonadotropins, estradiol-17β and progesterone concentrations. These results indicated that NGF and its receptors, TrkA and p75 may be involved in the regulation of seasonal changes in the ovarian functions of the wild ground squirrel.

## Introduction

The nerve growth factor (NGF) belongs to a family of related proteins required for the survival, maintenance, and development of discrete neuronal populations in the central and peripheral nervous systems [1-3]. It is also believed that NGF not only has an effect on the nervous system, but also plays an important role in a variety of non-neuronal system, such as immune, cardiovascular and endocrine systems [4-6]. The effect of NGF has been shown to be mediated through specific membrane receptors high-affinity tyrosine kinase A (TrkA), which is responsible for its biological activities [7,8]. Furthermore, the effect of NGF is also mediated via low affinity receptor p75 that also functions as other neurotropins’ receptor [9]. When p75 and TrkA receptors are co-expressed, p75 increases the sensitivity of the TrkA receptor and its signaling efficiency [10,11].

It is now well known that NGF and its receptors are expressed in the mammalian ovary, including women [12-14], rats [14,15], golden hamsters [16-18], cows [19], sheep [20] and Shiba goats [21]. More and more evidences have indicated that NGF signaling plays a critical role in the development of mammalian ovary, oogenesis and folliculogensis [22-24], in an auto- and/or paracrine manner. In our previous studies of the golden hamsters, NGF and its two receptors TrkA and p75 were present in ovaries, oviducts and uteri, demonstrating that NGF, TrkA and p75 have important autocrine and paracrine regulatory roles in the function of reproductive organs during the estrous cycle [16,25,26]. Data to support this concept in wild animals, however, is very limited. To study the basic mechanisms of NGF regulation of ovarian function during the breeding and nonbreeding seasons within the annual reproductive cycle, the wild ground squirrel offers a useful model without any manipulations.

The wild ground squirrel (*Citellus dauricus* Brandt) is a typical seasonal breeder which has a strict and extremely compressed breeding period (for female individuals, it includes estrous period, pregnancy and birth) from April to May and a long period of sexual dormancy from June to the following March including a 6-month hibernation period [27]. The wild female ground squirrel exhibits estrus immediately after emergence from hibernation in spring, and has a gestation period of 28 days [28]. Whether fertilized or not, all females become sexually inactive as the relatively brief breeding season ends. Previously, we observed the presence of inhibin/activin subunits in the ovary of the wild ground squirrels, indicating its important paracrine/autocrine regulatory role in the seasonal folliculogenesis of the wild ground squirrel [29]. In search for other key local players, we investigated the expression levels and immunolocalization of NGF and its receptors, TrkA and p75 in ovarian tissues of the wild ground squirrel during the breeding and nonbreeding seasons, and to elucidate the relationship between NGF and its receptors (TrkA and p75) and ovarian functions in this wild rodent.

## Material and methods

### Animals

All the procedures on animals were carried out in accordance with the Policy on the Care and Use of Animals by the Ethical Committee, Beijing Forestry University and approved by the Department of Agriculture of Hebei province, PR China (JNZF11/2007). Wild female ground squirrels that were regarded as adults according to their body weights (242–412 g) were captured on April 13 (10.2 hours of daylight) after emergence from hibernation in the breeding period (n = 10) and on June 9 (12.6 hours of daylight) in the nonbreeding period (n = 8) of 2009 in Hebei Province, PR China.

Animals were anesthetized with 4% isoflurane and blood samples were rapidly collected from leg vein. Plasma samples were frozen and stored at -20C, after the blood samples were added heparin sodium and centrifuged (3000 rpm, 20 min at 4C). An overdose of pentobarbital was applied afterwards for euthanasia. Ovary and brain were quickly removed and dissected. Length, width and weight of each ovary were measured. A part of the tissues were fixed in 0.05 M phosphate-buffered saline (PBS, pH 7.4) containing 4% paraformaldehyde for histological and immunohistochemical observation, while the rest were immediately frozen in liquid nitrogen and stored at -80C for RNA isolation and protein extraction.

### Histology

Ovarian samples were dehydrated in ethanol series and embedded in paraffin wax. Serial sections (4 μm) were mounted on slides coated with poly-L-lysine (Sigma, St. Louis, MO, U.S.A.). Sections were stained with hematoxylin-eosin (HE) for observations of general histology. The sections were screened using an Olympus photomicroscope with a × 20 objective lens and imaged with software Image-Pro Plus 4.5 (Media Cybernetics, Bethesda, MD, USA). Every one in ten serial sections, and altogether 50 and 30 sections for the breeding and nonbreeding season ovary respectively were selected for follicle identification [30] and quantification. Six random vision fields were selected per Section.

### Immunohistochemistry

Ovarian sections were blocked with 10% normal goat serum to prevent the non-specific binding of the second antibody. The sections were then incubated with polyclonal primary antibody against NGF (0.4 μg/ml, M-20), TrkA (2 μg/ml, 763) or p75 (2 μg/ml, H-92) (Santa Cruz Biotechnology, Santa Cruz, CA, USA) for 12 h at 4C, and incubated with the second antibody, goat anti-rabbit IgG conjugated with biotin and peroxidase with avidin for 1 h at room temperature. The sections were visualized using a rabbit ExtrAvidin™ staining kit (Sigma, St. Louis, MO, USA) in 150 ml of 0.05 M Tris–HCl buffer containing 30 mg 3,3-diaminobenzidine (Wako, Tokyo, Japan) plus 30 μl H_2_O_2_. Finally, the sections were counterstained with hematoxylin (Merck, Tokyo, Japan) and NGF, TrkA and p75 were detected, respectively. The immunostained slides were scanned using the software Image-Pro Plus 4.5 (Media Cybernetics, MD, USA) at 20× magnification. The specificity of NGF and its receptors, TrkA and p75 antibodies have been described previously [31]. The immunohistochemical staining was determined as positive (+), strong positive (++), very strong positive (+++), and negative (−). Staining that was weak but higher than control was set as positive (+); the highest intensity staining was set as very strong positive (+++); staining intensity between + and +++ was set as strong positive (++).

### Western blotting

Ovarian tissues were weighed and dissected into small pieces using a clean razor blade. The tissues were homogenized in a tissue homogenizer containing 300 μl of 10 mg/ml PMSF and incubated for 30 min on ice. Homogenates were centrifuged at 12,000 g for 10 min at 4C. Protein extracts (25 μg) were mixed with equal volumes of 2 × Laemmli sample buffer. Equal amounts of proteins from each sample were loaded onto a 12% SDS-PAGE gel and electrophoretically separated at 18 V/cm and transferred to nitrocellulose membrane using a wet transblotting apparatus (Bio-Rad, Richmond, CA, USA). The membrane was blocked in 3% BSA for 1 h at room temperature. Primary incubation of the membrane was carried out using NGF, TrkA or p75 antibody (1:1000 dilution) for 1 h at room temperature. Secondary incubation of the membrane was then carried out using an IRDye (1:5000 dilution, Rockland, Gilbertsville, PA, USA) for 1 h at room temperature. Finally, the membrane was washed in 25 ml Tris-Buffered Saline with Tween-20 (TBST wash buffer, 0.02 M Tris, 0.137 M NaCl and 0.1% Tween-20, pH 7.6) plus 3 μl H_2_O_2_ and visualized with Odyssey infrared imaging system. Brain tissue of wild ground squirrel was used as a positive control and water, instead of primary antisera, was used as a negative control. β-actin was selected as the endogenous control. The intensities of the bands were quantified using Quantity One software (Version 4.5, Bio-Rad Laboratories) and expression ratios were calculated.

### RNA isolation

Total RNA from each sample was extracted using ISOGEN (Nippon Gene, Toyama, Japan). Approximately 1 g of ovarian tissues were thawed and immediately homogenized in 10 ml of ISOGEN™. The homogenate was incubated for 5 min at room temperature to allow the complete dissociation of nucleoprotein complexes. After the addition of 2 ml of chloroform, the mixture was vigorously shaken for 3 min at room temperature and centrifuged at 12,000 g for 10 min at 4C. The aqueous phase was then transferred to a fresh tube and washed with an equal volume of chloroform. An equal volume of isopropanol was added, and the sample was kept for 10 min at room temperature. RNA was precipitated by centrifugation at 12,000 g for 10 min at 4C. The RNA pellet was washed twice with 75% ethanol, briefly dried under air, and dissolved in 100 μl of diethylprocarbonate-treated water.

### Reverse transcription-polymerase chain reaction (RT-PCR)

The first-strand cDNA from total RNA was synthesized using Superscript II Reverse Transcriptase (Invitrogen, Carlsbad, CA, USA) and oligo (dT)_12–18_ according to the manufacturer’s protocol. The 20 μl of reaction mixture contained 4 μg of total RNA, 0.5 μg of oligo (dT)_12–18_, 2.5 mM MgCl_2_, 0.5 mM dNTP, 10 mM dithiothreitol, 20 mM Tris–HCl (pH 8.4) and 200 U of Superscript II enzyme. The first-strand cDNA was used for PCR amplification with the appropriate primers previously proved (Table 1). Given the unknown genome of the wild ground squirrel, we could only design primers based on the sequence of mouse and rat [31], considering the relative conserved sequences between these rodents. The 100 μl of reaction mixture contained 1 μl of first-strand cDNA, 0.5 μM each primer, 1.5 mM MgCl_2_, 0.2 mM dNTP, 20 mM Tris–HCl (pH 8.4) and 2.5 U of *Taq* polymerase (Invitrogen, Carlsbad, CA, USA). The amplification was under the following condition: 94C for 5 min for the initial denaturation of the RNA/cDNA hybrid, 30 cycles of 94C for 1 min, 52C for 1 min, and 72C for 2 min for amplification. The PCR product was electrophoresed in the 2% agarose gel and individual bands were visualized by ethidium bromide staining. Brain tissue of wild ground squirrel was used as positive control and water, instead of cDNA, was used as negative control. The housekeeping gene, *RpL7*, was selected as the endogenous control as it is an estrogen-independent gene. The bands were quantified using Quantity One software (Version 4.5, Bio-Rad Laboratories) and expression ratios were calculated.

**Table 1:** Oligonucleotide primer sequences for PCR amplifications

**Primer**	**Sense**	**Antisense**
NGF	TCCACCCACCCAGTCTTC	GCTCGGCACTTGGTCTCA
TrkA	TCGGACCATGCTGCCCATCC	AGGCGTTGCTGCGGTTCTCG
p75	GGAGGACACGAGTCCTGAGC	CAGTGGAGAGTGCTGCAAAG
RpL 7	TCAATGGAGTAAGCCCAAAG	CAAGAGACCGAGCAATCAAG

### Cloning and sequencing of PCR products

The purified PCR products were ligated into pCR 2.1-TOPO (Invitrogen, Carlsbad, CA, USA) and the ligation products were used to transform the competent *E. coli* using TOPO TA Cloning Kit (Invitrogen, Carlsbad, CA, USA). Plasmids were extracted from the bacteria and positive clones containing the proper insert were sequenced in both directions using Thermo Sequenase II Dye Terminator Cycle Sequencing Premix Kit (Amershan Pharmacia Biotech, UK) with an automatic sequencing system (ABI PRISM 377, Applied Biosystems Japan, Tokyo, Japan). After obtaining the sequence of each PCR product, we blasted with the known mRNA sequences of mouse (NGF, NM_013609.3; TrkA, NM_001033124.1; p75, NM_033217.3; RpL7, NM_011291.5), rat (NGF, NM_001277055.1; TrkA, NM_021589.1; p75, NM_012610.2; RpL7, NM_001100534.1), bovine (NGF, NM_001099362.1; TrkA, XM_005898751.1; p75, NM_001102478.2; RpL7, NM_001014928.1) and human (NGF, NM_002506.2; TrkA, AB019488.2; p75, NM_002507.3; RpL7, NM_000971.3), find the homologous sequence fragments in each species and compare for homology using DNAman.

### Hormone assays

Plasma concentrations of estradiol-17β and progesterone were determined by double-antibody RIA systems using ^125^I-labeled radioligands as described previously [32]. Antisera against estradiol-17β (GDN 244) [33] and progesterone (GDN 337) [34] was kindly provided by Dr. G. D. Niswender (Animal Reproduction and Biotechnology, Colorado State University, Fort Collins, CO). The intra- and inter-assay coefficients of variation were 3.7% and 6.2% for estradiol-17β and 6.3% and 15.4% for progesterone, respectively. Plasma concentrations of follicle stimulating hormone (FSH) and luteinizing hormone (LH) were measured by double-antibody RIA systems using a rabbit antiserum against human FSH (#6; provided by NIDDK NIH, Bethesda, MD, USA) and a rabbit antiserum against ovine LH (YM #18; provided by Dr Y. Mori, Laboratory of Veterinary Ethology, University of Tokyo, Tokyo, Japan). The intra- and inter-assay coefficients of variation were 9.2% and 13.2% for FSH and 8.8% and 13.0% for LH, respectively.

### Statistical analysis

Means and standard deviations were calculated. Data were analyzed using a one-way ANOVA and the means were compared for significance using Duncan’s Multiple Range Test (P = 0.05) using the SPSS computer software package.

## Results

### The distinct variation in ovarian histology and folliculogenesis

**Figure 1:**
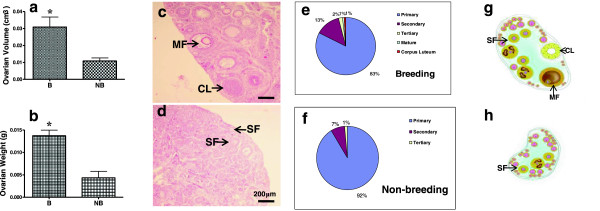
**Morphological and histological features of ovarian tissues of the wild ground squirrel during the breeding and nonbreeding periods.** Marked seasonal differences were observed in ovarian volume **(a)** and weight **(b)**. Subsequently, HE staining was performed for the ovaries of the breeding season **(c)** and nonbreeding season **(d)**, and follicles of the two periods were manually quantified accordingly **(e and f)**. **g** and **h** were the schematic diagrams for the varied folliculogenesis of the breeding and nonbreeding periods. B, breeding season; NB, nonbreeding season; MF, mature follicle; CL, corpus luteum; SF, secondary follicle. *, P < 0.01.

Ovaries of breeding and nonbreeding seasons were observed morphologically and histologically (Figure 1). In line with our previous reports, both the ovarian volume and weight were markedly higher in the breeding season than in the nonbreeding season (Figure 1a and b, p < 0.01). All levels of follicles, as well as the corpora lutea, were seen in the breeding season ovary (Figure 1c), whereas primary and secondary follicles comprised most of the nonbreeding season ovary, with few tertiary and mature follicles (Figure 1d). Based on the HE staining of the serial sections, we also quantified the numbers of different levels of follicles and corpora lutea (Figure 1e and f). Apparently, the lower ratio of primary follicles and higher ratios of secondary, tertiary, mature follicles and corpora lutea implied a more active follicuologenesis in the breeding period ovary. The distinct folliculogenesis of the breeding and nonbreeding seasons were schemed respectively in Figure 1g and h.

### The ovarian immunoreactivity of NGF, TrkA and p75 changes seasonally

**Figure 2:**
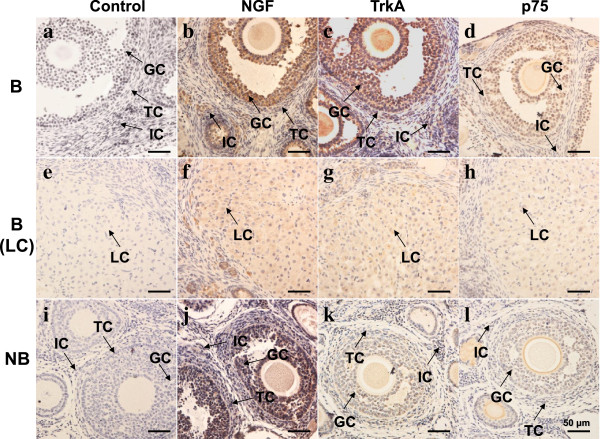
**Immunohistochemical localization of NGF ligand and receptors in ovaries of the wild ground squirrel during breeding and nonbreeding periods.** The first column **(a, e, i)** represents negative control samples of the breeding season ovary, corpus luteum and nonbreeding season ovary; the second column **(b, f, j)**, the third column **(c, g, k)**, and the fourth column **(d, h, l)** represent immunostaining with antibodies against NGF, TrkA and p75, in the breeding period ovary, corpus luteum and nonbreeding period ovary. B, breeding period; NB, the nonbreeding period; GC, granulosa cells; TC, theca cells; IC, interstitial cells; LC, luteal cells. Scale bar, 50 μm.

Immunohistochemistry was performed to detect the localization pattern of NGF and its receptors in the wild ground squirrel ovary and representative stainings were shown in Figure 2. In the breeding season, both NGF ligand and TrkA receptor were present in various types of cells, including the granulosa cells, theca cells, interstitial cells and corpora lutea, with the highest intensity in the granulosa cells (Figure 2b,c,f,g). Strong positive signals of p75 were also detected in these somatic cells, but not in the corpora lutea (Figure 2d and h). The immunostaining intensity generally decreased when it came to the nonbreeding season. NGF and TrkA were still positively stained in the secondary follicles of the nonbreeding season ovary, where mild signals of p75 were detected only in the granulosa and theca cells (Figure 2j,k,l). No signal was seen in the negative control panel (Figure 2a,e,i). The immunoreactivity of each staining was quantified and summarized in Table 2.

**Table 2:** Relative abundance of NGF, TrkA, and p75 in ovaries of the wild ground squirrels during the breeding and nonbreeding seasons

	**NGF**		**TrkA**		**p75**	
	B	NB	B	NB	B	NB
Granulosa cells	+++	++	+++	+	++	+
Thecal cells	++	+	++	+	++	+
Interstitial cells	++	+	++	+	+	-
Lutein cells	++	/	++	/	-	/

Immunoreactivity was shown as - for negative staining, + for the positive staining, ++ for the strong positive staining, +++ for the very strong positive staining and/for no such cell type.

### The seasonal changes in ovarian protein and mRNA expressions of NGF, TrkA and p75

**Figure 3:**
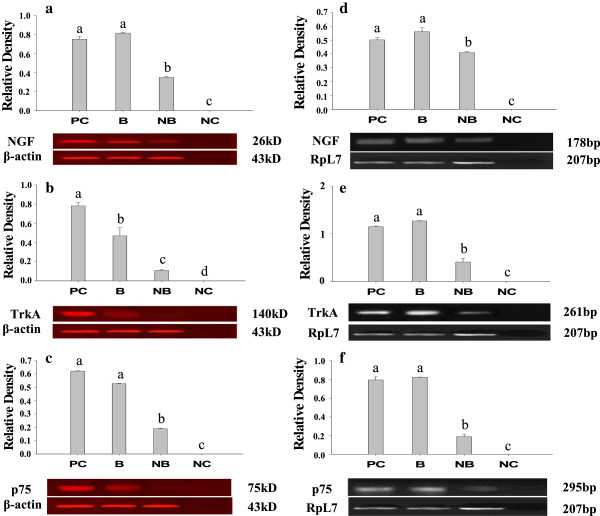
**Western blot and RT-PCR detections of NGF, TrkA and p75 in ovarian tissues of the wild ground squirrel during the breeding period (B) and nonbreeding period (NB), respectively.** The left column shows the western bolt results of NGF **(A)**, TrkA **(B)** and p75 **(C)** respectively, The right column shows the RT-PCR results of NGF **(D)**, TrkA **(E)** and p75 **(F)** respectively. The proteins extracted from brains were used as the positive control (PC). Water was used as the negative control (NC). β-actin blots shown were used as controls to correct for loading in each lane. The expression levels were determined by densitometric analysis. Bars represent means + SD for five independent experiments. Means within the columns marked with different letters indicate significant difference (P < 0.01).

We then moved on to detect the protein and mRNA expression levels of NGF, TrkA and p75 using Western blot and PCR, and representative bands were shown in Figure 3. The intensity of each band was normalized to the level of β-actin and RpL7, used as the endogenous control, for Western and PCR detections respectively. Both the protein and mRNA expression levels of NGF ligand and receptors were significantly higher in the breeding season when compared to the nonbreeding season, basically in consistent with the immunohistochemical results (Figure 3, p < 0.01). Notably, the relative protein level of NGF and the relative mRNA levels of NGF, TrkA and p75 were even higher in the breeding season ovary than in the brain (P < 0.05), reassuring the substantial role of NGF during this particular time.

To further confirm the nature of the PCR signals, cDNA fragments of NGF, TrkA and p75 in ovarian tissues were sequenced and compared to the corresponding fragments in mouse, rat, bovine, and human. The partial mRNA sequences in the wild ground squirrel are as below:

NGF#

GGGGGACTCAGTGTGTGTGCTGGTGTCAGTGTGTGGGTTGGAGATAAGACCACAGCCACAGACATCAAGGGCAAGGAGGTGACAGTGCTGGCCGAGGTGAACATTAACAACAGTGTATTCAGACAGTACTTTTTTGAGACCAAGTGCCGAGC

TrkA#

GGAAGTGACATCTCTACCGCAGTTCAGCACCGAGAGCGATGTGTGGAGCTTTGGGGTGGTGCTCTGGGAGATCTTCACCTATGGAAAGCAGCCCTGGTACCAGCTCTCTAACACTGAGGCGATCGAGTGTATCACGCAGGGCCGGGAGCTGGAGCGGCCGCGCGCCTGCCCTCCTGATGTCTACGCCATCATGCGAGGCTGCTGGCAGCGAGAACCGAATCAACGCCT

P75#

CCAGTAGGGCAGTGTGGCGGAGCCTTGCGGAGCCATCCAGACCGTGTGTGAACCCTGCCTGGACAGTGTTACGTTCTCTGACGTGGTGAGCGCCACCGAGCCGTGCAAGCCGTGCACCGAGTGCCTGGGCCTGCAGAGTATGTCCGCTCCCTGTGTGGAGGCAGACGATGCCGTGTGCCGATGCTCCTATGGCTACTACCAGGACGAGGAGACTGGCCGCTGCGAGGCTTGCAGCGTGTGCGGGGTGGGCTCAGGATCGGTGTCCTCCC

RpL7#

AAGGATCTGCTGCTGCTTCTGTTCCAGATCTCAATGGCACCTTTGTTAAGCTCAACAAGGCTTCAATTAACATGCTGCGGATTGTGGAGCCATACATTGCATGGGGGTACCCCAACCTGAAGTCAGTAAACGAGCTCATCTACAAGCGAGGCTACGGCAAAATCAACAAGAAGCGGATTGCCTTGACAGATAATTCCTTGAATGCACGGTCTCTT

The 176-bp NGF cDNA nucleotide sequence identity was 92.11%, 90.20%, 81.70% and 86.18%, respectively; the 261-bp TrkA cDNA nucleotide sequence identity was 94.32%, 90.83%, 84.72% and 83.41%, respectively; the 295-bp p75 cDNA nucleotide sequence identity was 94.42%, 88.10%, 84.01% and 83.64%, respectively; the 207-bp RpL7 cDNA nucleotide sequence identity was 94.55%, 84.65%, 74.23% and 76.87%, respectively (Table 3), which not only confirmed the specificity of PCR primers but also suggested high affinities of NGF, TrkA and p75 genes between the wild ground squirrel and the species compared.

**Table 3:** Nucleotide sequence identity in ovarian tissues of wild ground squirrel in comparison with mouse, rat, bovine and human (%)

	**NGF**	**TrkA**	**p75**	**RpL7**
Mouse	92.11	94.32	94.42	94.55
Rat	90.20	90.83	88.10	84.65
Bovine	81.70	84.72	84.01	74.23
Human	86.18	83.41	83.64	76.87

### Plasma concentration of LH, FSH, estradiol-17β and progesterone

**Figure 4:**
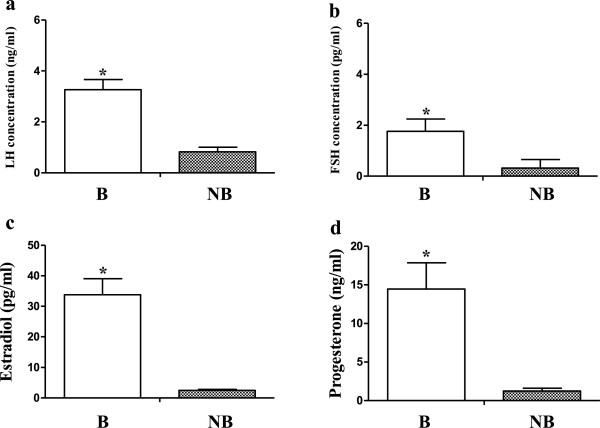
**Seasonal change of the plasma concentration of LH, FSH, estradiol-17β and progesterone.** Plasma LH **(a)**, FSH **(b)**, estradiol-17β **(c)**, and progesterone **(d)** levels in the wild ground squirrel during breeding period (B) and nonbreeding period (NB). Bars represent means + SD for five independent experiments. Means within the columns marked with different letters indicate significant difference (P < 0.01).

The profiles of plasma LH, FSH, estradiol-17β and progesterone are shown in Figure 4. Plasma LH, FSH, estradiol-17β and progesterone concentrations were all remarkably higher in the breeding season as compared to the nonbreeding period (Figure 4a,b,c,d, p < 0.01).

## Discussion

The present study demonstrated that immunoreactivities of NGF and its two receptors, TrkA and p75 were greater in the ovaries of the breeding season then decreased to a relatively low level in the nonbreeding season, and the expression patterns of NGF ligand and receptors were correlated with the changes of plasma concentrations of gonadotropins, estradiol-17β and progesterone. These findings suggested that NGF, TrkA and p75 may be involved in the regulation of seasonal changes in the ovarian functions of the wild ground squirrels.

The present histological result was in agreement with previous data reported in this species, that the number of primary follicles had no significant difference between the breeding and nonbreeding seasons, whereas the number of secondary follicles, antral follicles, post-antral follicles and corpus luteum displayed a significantly decrease from the breeding season to the nonbreeding season [28,29]. It suggested that primary follicles were stopped from developing to the stage of secondary follicles, antral follicles, post-antral follicles during nonbreeding season in the wild ground squirrel ovary. The fact that corpus luteum was not discovered in the nonbreeding season strongly suggested that no ovulation occurred in this stage [28]. In the present study, NGF and its receptors, TrkA and p75 were immunolocalized in ovarian tissues during the breeding and nonbreeding period, and both mRNA and protein of NGF, TrkA and p75 were also detected, indicating *in situ* synthesis and secretion of NGF and its receptors in ovaries of the wild ground squirrel, where the role of NGF might be mediated via both receptors to affect follicular development. Moreover, the expression levels of NGF and its receptors, TrkA and p75 were significantly higher in the breeding season as compared to the nonbreeding season, implying that NGF system may be involved in the regulation of ovarian function change. Our findings in the wild ground squirrel was generally in line with previous reports in pig and ewe, which showed higher contents of NGF and TrkA in large follicles than in smaller follicles [20,35]. Given the increased expression of NGF and its receptors in ovary of the breeding season, as well as the distinct morphological change, it is reasonable to postulate that NGF contributes to not only the innervation of rapidly growing follicles, but also steroidogenic cell proliferation and steroid production in the breeding season ovary, as has been observed in other rodents [16,36,37].

The female reproductive system undergoes a number of programmed cyclical processes during the course of the ovulatory cycle. NGF and its receptors, under the influence of gonadotropins and/or ovarian hormones, may play a crucial regulatory role in these processes [25]. In the present study, the expression of NGF and its receptors were correlated with changes in plasma concentrations of LH, FSH, estradiol-17β and progesterone during the breeding and nonbreeding seasons. These results were similar to those found in golden hamsters and pigs. Previous studies in golden hamster have suggested that LH surge may be an important factor for inducing the expression of NGF, TrkA, p75 in ovarian tissues periodically [16,18]. In pig ovary, the increase in expression of NGF and TrkA in large follicles found on day 20 may also result from an LH effect [35]. Dissen *et al.* reported that in juvenile rats treated with equine chorionic gonadotropin, significantly elevated TrkA mRNA levels were found in ovary after the first preovulatory LH surge [38]. Similarly, the functional relationship between gonadotropins and NGF in studies of the sheep ovary showed that large follicles respond with increased NGF release to *in vitro* stimulation with a combination of LH and FSH [20]. Earlier studies also indicated that NGF activity and content were found to be increased by estradiol in a glioma cell line culture [39]. In addition, our previous studies showed that the expression of NGF, TrkA and p75 in the uterus of the wild ground squirrel was highest in the breeding season, when estradiol and progesterone production were greatest [31]. Taken together, the present results are in accordance with the views that NGF and its receptors are expressed in ovarian tissues, and these expressions are gonadotropins and/or ovarian hormones-dependent, and therefore, may contribute to events either led to or associated with the ovulatory process [13].

The present study showed that immunolocalization for NGF and its receptors were also found in luteal cells during the breeding season, which was similar to those observed in other species such as golden hamster [16], Shiba goat [21] and porcine [35]. In golden hamster, luteal cells displayed a stronger reaction for NGF and its receptors in metestrus than in estrus and diestrus [16]. In pig, the increased NGF and TrkA protein levels in luteal cells were found during the estrous cycle [35]. These results shed light to the critical role of NGF in development and/or maintenance of luteal function, which is further supported by the findings that NGF can stimulate of progesterone and oxytocin release [40] and acetylcholine production [41] in bovine luteal cells as well as maintenance of luteal vasculature in rats [37]. In line with these aforementioned data, our present results indicated that NGF ligand and receptors might play a similar role in the development of corpora lutea in the wild seasonally-breeding rodent.

Cellular growth is related to the ability to promote proliferation of mesenchyme and follicular cells, as well as to induce FSHR synthesis [13,15]. In our previous studies, the Western blotting results of FSHR showed that significant induction in the breeding season compared with the nonbreeding season [28]. This study revealed that the change levels of gonadotropins were parallel to those in the expression patterns of ovarian NGF and receptors during the breeding and the nonbreeding seasons. In rats, ovaries treated with NGF developed the capacity to response to FSH, with the formation of cAMP in preantral follicles [15]. Similar results were obtained in human cells, in which the culture of granulosa cells with NGF also increased expression of the FSHR in these cells [13]. These results implied that NGF may act indirectly in follicular development through the production of biologically active FSHR [13]. In golden hamsters, as one member of TGFβ superfamily, inhibin α-subunit might works in concert with NGF and its receptors to act on LH/hCG receptor expression in ovarian interstitial cells and associated with the ovulatory process [18]. Also, our previous studies showed that inhibin/activin subunits (α, β(A) and β(B)) were present in granulosa cells, theca cells of antral follicles and interstitial cells in the breeding season ovary of wild ground squirrel, following ovulation, the corpora lutea become a major site of inhibin/activin synthesis [29]. Moreover, the expression patterns of inhibin/activin subunits in the wild ground squirrel ovary were also consistent with the present results. Thus, combined with previous reports, this study suggested that the co-expression of NGF and its receptors, along with other key growth factors, including inhibin/activin subunits, might indicate synergistic actions of them in the regulation of seasonal folliculogenesis in the wild ground squirrel.

In conclusion, we have shown, for the first time, the expression patterns of ovarian NGF, TrkA and p75 during the breeding and nonbreeding seasons in the wild ground squirrel. Our results revealed a close correlation between NGF expression and gonadotropins and steroid hormones, which implicated that NGF ligand and receptors are likely to be involved in the regulation of seasonal changes in the ovarian functions of the wild ground squirrel. The interesting alterations in ovarian morphology and histology as well as folliculogenesis observed between breeding and nonbreeding periods in the wild ground squirrel demonstrate a complex regulatory mechanism (s) that involves both sex steroids and growth factors.

## Competing interests

The authors declare that they have no competing interests.

## Authors’ contributions

BL participated in performing the experiments, analyzing the data and drafting the manuscript. XS, LB, SH, QL and YL assisted with sample collection, all experiments and helped revising the manuscript. YH and QW designed, supervised the study, and revised manuscript. GW and KT provided some reagents and revised the manuscript. All authors read and approved the final manuscript.
